# ESRP1-Associated CD44 Alternative Splicing Stratifies Epithelial–Mesenchymal Identity States in a Non-Transformed Human Cell System

**DOI:** 10.3390/cimb48020130

**Published:** 2026-01-24

**Authors:** Karolina Bajdak-Rusinek, Natalia Diak, Anna Trybus, Agnieszka Fus-Kujawa, Marcelina Salamon, Jan Olszewski, Weronika Wójtowicz, Patrycja Rozwadowska-Kunecka

**Affiliations:** 1 Department of Molecular Biology, Faculty of Medical Sciences in Katowice, Medical University of Silesia, Medykow 18 Street, 40-752 Katowice, Poland; natalia.jarosz@sum.edu.pl (N.D.); anna.trybus@sum.edu.pl (A.T.); afus@sum.edu.pl (A.F.-K.); s88614@365.sum.edu.pl (M.S.); s88125@365.sum.edu.pl (J.O.); s83238@365.sum.edu.pl (W.W.); prozwadowska@uck.gda.pl (P.R.-K.); 2 Students Scientific Society, Faculty of Medical Sciences, Medical University of Silesia, 40-055 Katowice, Poland; 3 Doctoral School of the Medical University of Silesia in Katowice, 40-055 Katowice, Poland; 4 Department of Pediatrics, Diabetology and Endocrinology, University Clinical Center, 80-952 Gdańsk, Poland

**Keywords:** epithelial–mesenchymal transition (EMT), CD44 isoform switching, ESRP1, epithelial–mesenchymal plasticity, induced pluripotent stem cells (iPS cells), iPS-derived mesenchymal stem cells (iPS-MSCs), alternative splicing

## Abstract

Epithelial–mesenchymal plasticity encompasses a spectrum of epithelial and mesenchymal identity states that enable cells to adapt to changing biological contexts. While CD44 isoform usage and epithelial splicing regulators ESRP1/2 are well-characterized in cancer-associated epithelial–mesenchymal transition (EMT), their regulation across physiological, non-transformed identity states remains less well defined. Here, we employed a non-malignant human cellular system comprising primary dermal fibroblasts, induced pluripotent stem (iPS) cells, and iPS-derived mesenchymal stem cells (iPS-MSCs) to define discrete epithelial, intermediate epithelial/mesenchymal, and mesenchymal identity states positioned along an epithelial–mesenchymal identity axis. Morphological assessment, lineage marker profiling, and RT-qPCR analyses revealed reproducible population-level stratification of these states. CD44 expression and alternative splicing followed this hierarchy, with CD44s predominating in fibroblasts, broad variant exon inclusion in iPS cells, and intermediate patterns in iPS-MSCs. ESRP1 expression mirrored CD44 splicing architecture, and ESRP1 silencing in iPS cells induced a shift toward CD44s, confirming its functional contribution to epithelial-associated CD44 splicing. In contrast, Notch-related transcriptional readouts displayed distinct, context-dependent profiles across the examined identity states. Together, this study establishes a tractable non-transformed human model that captures selected molecular features associated with epithelial–mesenchymal plasticity beyond malignant contexts.

## 1. Introduction

Epithelial–mesenchymal transition (EMT) and its reverse process, mesenchymal–epithelial transition (MET), are fundamental biological programs that enable cells to alter their identity, respond to environmental cues and acquire new functional states. Rather than representing binary switches, these processes generate a spectrum of epithelial (E), intermediate epithelial/mesenchymal (E/M), and mesenchymal (M) phenotypes, collectively contributing to cellular plasticity. In cancer, such plasticity underpins tumor progression, invasion, metastatic dissemination, and therapy resistance, making the regulation of EMT/MET a central focus in efforts to understand the molecular drivers of metastasis and identify potential therapeutic vulnerabilities [1,2,3,4].

Among the molecular features associated with EMT-related plasticity, the CD44 adhesion receptor occupies a prominent position. The CD44 gene undergoes extensive alternative splicing to generate the standard isoform CD44s and multiple epithelial-associated variant isoforms (CD44v). A large body of evidence demonstrates that CD44 isoform usage correlates with transitions along the E/M axis: CD44s is mostly enriched in mesenchymal and invasive states, whereas CD44v isoforms are associated with epithelial and stem-like phenotypes [5,6,7]. This splicing switch is governed primarily by the epithelial splicing regulators ESRP1 and ESRP2, which promote the inclusion of variant exons and thereby sustain epithelial identity. Downregulation of ESRP1/2 is a characteristic molecular feature of EMT and favors a CD44s-dominant profile linked to enhanced cellular motility [8,9,10].

Multiple upstream signaling pathways converge on EMT programs, including TGF-β, Wnt, and Notch signaling. In malignant contexts, these pathways have been shown to modulate epithelial and mesenchymal gene expression programs, in part through transcriptional repression of epithelial regulators such as ESRP1 and ESRP2, thereby indirectly influencing alternative splicing patterns, including CD44 isoform selection [11,12]. While these regulatory relationships have been extensively characterized in cancer, it remains unclear as to what extent core splicing-based mechanisms of epithelial–mesenchymal plasticity are preserved during physiological, non-transformed cell identity transitions. Non-malignant cellular systems provide a powerful framework for dissecting the core mechanisms underlying cell-state plasticity without the confounding influence of oncogenic mutations and tumor-associated epigenetic instability. Importantly, however, such non-transformed systems have rarely been exploited to systematically map alternative splicing architectures associated with epithelial–mesenchymal identity states, particularly with respect to CD44 isoform regulation and its splicing control by ESRP proteins. In this regard, primary human fibroblasts, induced pluripotent stem (iPS) cells, and iPS-derived mesenchymal stem cells (iPS-MSCs) represent three well-defined and experimentally accessible cell identities. Fibroblasts display classical mesenchymal morphology and transcriptional profiles, whereas iPS cells form compact epithelial colonies characteristic of pluripotent states. iPS-MSCs, generated from iPS cells through directed differentiation, exhibit mesenchymal features while retaining aspects of their pluripotent origin at the transcriptional level. Notably, somatic reprogramming itself is driven by a MET process that mirrors key elements of EMT/MET transitions observed during development and cancer progression [13,14,15]. Together, this system provides a controlled and physiologically relevant framework for exploring molecular events associated with transitions along the E/M axis, independent of malignant transformation [16,17].

Here, we establish a non-transformed human benchmark system to investigate ESRP1-mediated CD44 alternative splicing across defined epithelial, intermediate, and mesenchymal identity states. By decoupling epithelial–mesenchymal plasticity from oncogenic transformation, this system allows for the assessment of fundamental, physiological splicing regulation associated with cell-state identity. Our aim was to determine whether these cell states recapitulate splicing-based regulatory patterns known to accompany EMT in cancer while disentangling core identity-associated mechanisms from context-dependent signaling pathways. Using an integrated analysis of CD44 alternative splicing and ESRP expression, we describe coordinated, identity-associated patterns that stratify epithelial, intermediate, and mesenchymal states along the E/M axis.

## 2. Materials and Methods

### 2.1. Generation of iPS Cells

Primary human neonatal dermal fibroblasts were obtained from the American Type Culture Collection (ATCC, Manassas, VA, USA; Cat. No. PCS-201-010) and cultured according to the supplier’s instructions provided by ATCC. Cells were used for reprogramming between passages 3 and 6 and routinely tested negative for mycoplasma contamination. Fibroblasts were reprogrammed into induced pluripotent stem (iPS) cells using the feeder-free CytoTune™ iPS 2.0 Sendai Reprogramming Kit (Thermo Fisher Scientific, Waltham, MA, USA), according to the manufacturer’s instructions. Briefly, fibroblasts were seeded two days prior to transduction onto 6-well plates at a density of 5 × 10^4^ cells per well in standard fibroblast culture medium. Reprogramming was initiated by transduction with Sendai viral vectors encoding the four Yamanaka factors OCT3/4, SOX2, KLF4, and c-MYC. Seven days after transduction, cells were transferred onto vitronectin-coated plates (5 µg/mL; Thermo Fisher Scientific), and the culture medium was replaced with Essential 8 (E8) medium (Life Technologies, Waltham, MA, USA, Cat No. A1517001) on the following day. Emerging iPS cell colonies were monitored for 3–4 weeks. Individual colonies were manually selected and expanded to prevent spontaneous differentiation and overconfluency. All procedures were performed in strict accordance with the manufacturer’s protocol.

### 2.2. Differentiation of iPS Cells into Mesenchymal Stem Cells

Mesenchymal stem cells derived from iPS cells (iPS-MSCs) were generated using the STEMdiff™ Mesenchymal Progenitor Kit (StemCell Technologies, Vancouver, BC, Canada). Differentiation procedures were performed exactly as specified by the manufacturer, including medium changes, induction steps, and maintenance conditions, until a stable MSC-like population was obtained.

### 2.3. Flow Cytometry

Cells were fixed in 70% ethanol for 10 min at 37 °C and permeabilized in 90% methanol for 30 min on ice. After washing, cells were incubated with primary antibodies diluted in FACS buffer (FC001) for 30 min at room temperature, followed by incubation with fluorophore-conjugated secondary antibodies for an additional 30 min. After final washes, cells were resuspended in PBS and analyzed using a FACSAria I flow cytometer (BD Biosciences, Franklin Lakes, NJ, USA). At least 10,000 events were acquired per sample. Fluorescence signals were analyzed using unstained and isotype controls to establish gating strategies. The following antibodies were used: SSEA-1, SSEA-4 (1:200), TRA-1-60 (Alexa Fluor™ 488-conjugated, Thermo Fisher Scientific), TRA-1-81, CD73-FITC (1:20), CD90-FITC (1:5), and CD105-FITC (1:20) (STEMCELL Technologies, Cologne, Germany).

### 2.4. RNA Isolation and Quantitative RT-PCR

Total RNA was extracted using the RNeasy Plus Mini Kit (Qiagen, 74136, Venlo, The Netherlands) following the supplied protocol. First-strand cDNA synthesis was performed with the RevertAid First Strand cDNA Synthesis Kit (Thermo Fisher, K1622) using 1 μg of total RNA and both oligo (dT) and random primers.

Quantitative PCR reactions were set up with Power SYBR Green PCR Master Mix (Applied Biosystems, 4368702, Carlsbad, CA, USA) and primer pairs listed in Supplementary Table S1. Reactions included cDNA corresponding to 7.5 ng of input RNA and 300 nM primers and were run on a LightCycler 480 system (Roche, Basel, Switzerland). Primer specificity was verified by melt curve analysis and agarose gel electrophoresis. Gene expression levels were normalized to ACTB and calculated using the Pfaffl method [18].

### 2.5. siRNA Transfection

Transient gene silencing was performed using small interfering RNAs (siRNAs). Cells were seeded in 12-well plates and transfected with siRNAs targeting ESRP1 (Genomed, Warsaw, Poland; 100 pmol per well) using Oligofectamine™ Transfection Reagent (Invitrogen, Waltham, MA, USA) according to the manufacturer’s instructions. A non-targeting siRNA (Ambion, Foster City, CA, USA, Cat. No. 4390847) was used as a negative control. Cells were harvested 48 h post-transfection for downstream analyses. All experiments were performed using at least three independent biological replicates.

The siRNA sequence used for ESRP1 knockdown was:

5′-CACAAGCAGAGUAUUUA-3′.

### 2.6. Statistical Analysis

All quantitative data are presented as the mean ± standard deviation (SD) from at least three independent biological replicates. Statistical analyses were performed using Microsoft Excel. For comparisons between two groups, a two-tailed Student’s *t*-test was applied. For analyses involving more than two independent groups, one-way analysis of variance (ANOVA) was used where appropriate. Statistical significance thresholds were defined as * *p*  <  0.05; ** *p*  <  0.01; *** *p*  <  0.001; **** *p*  <  0.0001. Exact group comparisons are specified in the corresponding figure legends.

## 3. Results

### 3.1. Morphology, Lineage Markers and EMT Markers Define Three Distinct Epithelial–Mesenchymal Identity States

**Figure 1:**
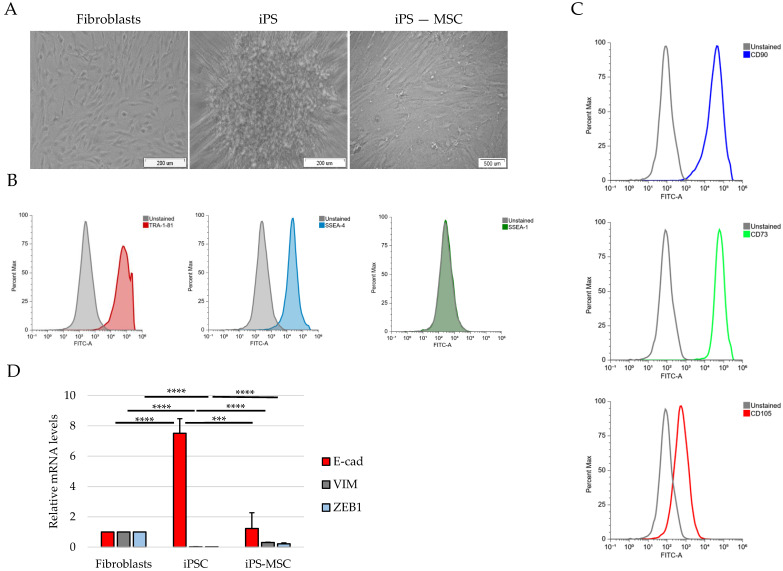
Morphological and phenotypic characterization of fibroblasts, iPS cells, and iPS-derived MSCs. (**A**) Phase-contrast images of human foreskin fibroblasts, iPS cell colonies, and iPS-derived mesenchymal stem cells (iPS-MSCs), (**B**) flow cytometry analysis of pluripotency markers (TRA-1-81, SSEA-4, SSEA-1) in iPS cells, (**C**) flow cytometry analysis of MSC markers (CD73, CD90, CD105) in iPS-MSCs, (**D**) RT-qPCR analysis of E-cad, VIM and the EMT-associated transcription factor ZEB1 expression in fibroblasts, iPS-MSCs, and iPS cells. Data are presented as the mean ± SD from three independent biological replicates. Statistical significance was determined as *** *p* < 0.001, **** *p* < 0.0001.

To characterize the epithelial–mesenchymal identity of the three non-transformed human cell populations, we examined their morphology, lineage marker expression and selected EMT-associated markers. These analyses revealed clear and consistent differences between primary dermal fibroblasts, iPS cells, and iPS-derived mesenchymal stem cells (iPS-MSCs). Fibroblasts displayed an elongated, spindle-shaped morphology, whereas iPS cells formed compact, epithelial-like colonies with high cell density. In contrast, iPS-MSCs exhibited a uniform fibroblastoid morphology characteristic of mesenchymal stem cells (Figure 1A).

Flow cytometry confirmed successful reprogramming of iPS cells, which expressed high levels of the pluripotency markers TRA-1-81 and SSEA-4, with SSEA-1 serving as a negative control (Figure 1B). Conversely, iPS-MSCs expressed canonical mesenchymal stem cell markers, including CD73, CD90 and CD105, confirming their MSC identity (Figure 1C).

To further define epithelial–mesenchymal status at the transcriptional level, we assessed the expression of the epithelial marker E-cadherin (E-cad), the mesenchymal marker Vimentin (VIM), and the EMT-associated transcription factor ZEB1 by RT-qPCR. iPS cells exhibited high E-cad and low VIM and ZEB1 expression, fibroblasts showed the opposite pattern, and iPS-MSCs displayed intermediate expression of all three markers, consistent with their positioning along the epithelial–mesenchymal (E/M) identity axis (Figure 1D).

Together, these morphological, phenotypic and transcriptional features establish a clear separation between fibroblasts, iPS cells, and iPS-derived MSCs, supporting their classification as mesenchymal, epithelial, and intermediate epithelial/mesenchymal identity states at the population level. These classifications reflect population-level molecular and phenotypic averages and do not imply uniform single-cell identity within each population.

**Figure 2:**
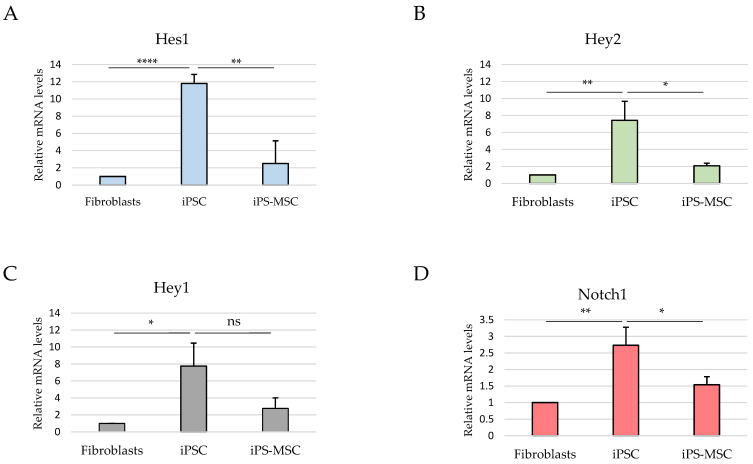
Notch-related transcriptional profiles across fibroblasts, iPS-derived MSCs, and iPS cells. RT-qPCR analysis of Notch1 and canonical downstream transcriptional targets HES1, HEY1, and HEY2 in primary dermal fibroblasts, induced pluripotent stem (iPS) cells, and iPS-derived mesenchymal stem cells (iPS-MSCs). Panels show relative mRNA expression levels of HES1 (**A**), HEY2 (**B**), HEY1 (**C**), and NOTCH1 (**D**), normalized to ACTB and presented as the mean ± SD from at least three independent biological replicates. Statistical significance is indicated directly on the graphs and refers to pairwise comparisons between iPS cells and fibroblasts and/or between iPS cells and iPS-MSCs, as specified for each gene. * *p* < 0.05, ** *p* < 0.01, **** *p* < 0.0001; ns, not significant.

To provide broader signaling context for the defined epithelial–mesenchymal states, we analyzed the expression of selected components of the Notch signaling pathway and downstream transcriptional targets by RT-qPCR (Figure 2). These analyses revealed distinct and context-dependent Notch-related transcriptional profiles across fibroblasts, iPS-MSCs, and iPS cells; however, these data represent mRNA-level readouts and do not directly assess functional Notch pathway activity.

### 3.2. CD44 Expression and Exon-Level Splicing Patterns Across the Epithelial–Mesenchymal Spectrum

**Figure 3:**
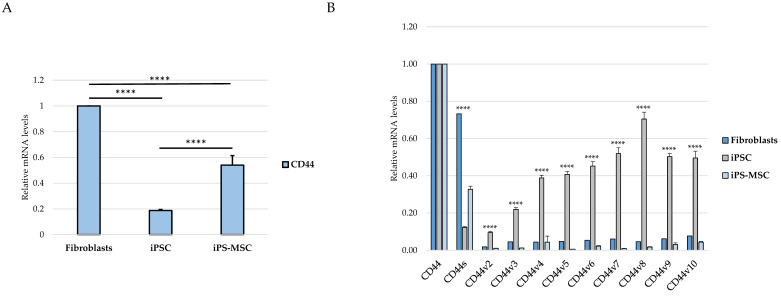
CD44 expression and isoform distribution across the fibroblast–iPS-MSC–iPS axis. (**A**) RT-qPCR analysis of total CD44 mRNA expression in primary dermal fibroblasts, induced pluripotent stem (iPS) cells, and iPS-derived mesenchymal stem cells (iPS-MSCs). (**B**) RT-qPCR analysis of CD44 variant exon inclusion (v2–v10), assessed by exon-specific primers, in fibroblasts, iPS cells, and iPS-MSC. Data are shown as relative mRNA levels normalized to ACTB and presented as the mean ± SD from at least three independent biological replicates. Asterisks indicate statistically significant differences observed in predefined pairwise comparisons between iPS cells and the other cell states (fibroblasts and iPS-MSCs). For all indicated comparisons, **** *p* < 0.0001.

We next quantified total CD44 transcript levels across the three cell types. RT-qPCR analysis revealed that primary dermal fibroblasts expressed the highest levels of total CD44, whereas iPS cells exhibited markedly reduced expression. iPS-derived MSCs displayed intermediate CD44 levels between fibroblasts and iPS cells, indicating that overall CD44 expression progressively decreases along the relative mesenchymal-to-epithelial identity axis represented by fibroblasts, iPS-MSCs, and iPS cells (Figure 3A).

To determine whether this global trend was accompanied by changes in CD44 splicing architecture, we next analyzed the relative inclusion of CD44 standard and variant-containing transcripts. Fibroblasts exhibited a CD44 expression profile dominated by the standard isoform, with low relative inclusion of variant exons v2–v10 as assessed by exon-specific RT-qPCR. In contrast, iPS cells exhibited robust expression of multiple CD44 variant-containing transcripts, consistent with a splicing pattern characteristic of epithelial identity. iPS-derived MSCs displayed intermediate expression levels across nearly all examined variant exons, falling between fibroblasts and iPS cells (Figure 3B).

Together, these data demonstrate that both total CD44 expression and CD44 alternative splicing pattern change in a coordinated manner across the epithelial–mesenchymal identity axis, enabling transcriptomic stratification of fibroblasts, iPS-MSCs, and iPS cells into mesenchymal, intermediate, and epithelial identity categories.

### 3.3. ESRP1 Expression Is Associated with CD44 Isoform Composition

**Figure 4:**
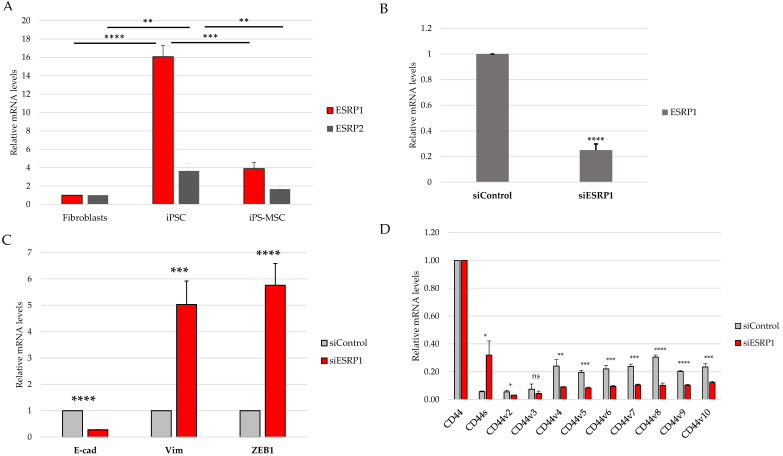
ESRP1 expression and functional contribution to CD44 splicing patterns. (**A**) RT-qPCR analysis of ESRP1 and ESRP2 expression in primary dermal fibroblasts, iPS-derived mesenchymal stem cells (iPS-MSCs), and iPS cells. (**B**) RT-qPCR confirmation of ESRP1 knockdown in iPS cells following transient siRNA-mediated silencing (siESRP1) compared with non-targeting control siRNA (siControl). (**C**) RT-qPCR analysis of selected epithelial–mesenchymal transition-associated markers (E-cadherin, Vimentin, ZEB1) in iPS cells after ESRP1 silencing. (**D**) RT-qPCR analysis of CD44 standard (CD44s) and variant exon–containing transcripts (v2–v10) in iPS cells following ESRP1 knockdown, demonstrating a shift from a CD44v-enriched to a CD44s-dominant splicing pattern. Data are shown as relative mRNA levels normalized to ACTB and presented as the mean ± SD from at least three independent biological replicates. Statistical significance is indicated as * *p* < 0.05, ** *p* < 0.01, *** *p* < 0.001, **** *p* < 0.0001.

To assess whether differences in CD44 isoform usage correlate with the expression of epithelial splicing regulators, we first analyzed ESRP1 and ESRP2 mRNA levels across the three cell states. RT-qPCR analysis revealed a strong enrichment of ESRP1 expression in iPS cells, markedly lower levels in primary human foreskin fibroblasts, and intermediate expression in iPS-derived MSCs. ESRP2 exhibited a similar, albeit less pronounced expression pattern (Figure 4A).

Based on its higher expression in epithelial iPS cells and its well-established dominant role in regulating epithelial-specific alternative splicing, ESRP1 was selected for functional perturbation in this study. To directly evaluate the contribution of ESRP1 to CD44 isoform selection, we next performed transient ESRP1 knockdown in iPS cells, which exhibited the highest basal ESRP1 expression. Efficient silencing of ESRP1 transcripts was confirmed by RT-qPCR (Figure 4B).

ESRP1 depletion resulted in a consistent reduction in CD44 variant exon-containing transcripts, accompanied by a reciprocal increase in CD44s expression (Figure 4C).

Together, these results demonstrate that ESRP1 directly contributes to the maintenance of an epithelial-associated, CD44v-enriched splicing pattern in iPS cells.

### 3.4. Integrated Trajectory of E/M Plasticity—Graphical Model

**Figure 5:**
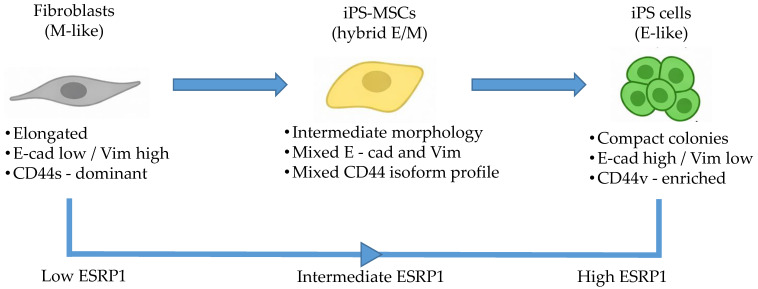
Integrated graphical model of E/M plasticity and CD44 splicing across non-transformed human cell states. The schematic summarizes the relative positioning of primary dermal fibroblasts, iPS-derived mesenchymal stem cells (iPS-MSCs), and iPS cells along an epithelial–mesenchymal (E/M) identity axis. The horizontal axis reflects cellular identity states rather than experimental lineage progression. Although fibroblasts are experimentally reprogrammed into iPS cells and iPS-MSCs are subsequently derived from iPS cells, the diagram maps these populations according to their molecular and phenotypic characteristics, including morphology, EMT marker expression, CD44 isoform usage, and ESRP1 expression. In this framework, fibroblasts represent a mesenchymal state, iPS cells an epithelial pluripotent state, and iPS-MSCs represent an intermediate epithelial/mesenchymal identity defined by population-level molecular and phenotypic features. This designation reflects averaged marker expression across cell populations and does not imply a homogeneous or stable hybrid EMT phenotype at the single-cell level.

To synthesize our findings, we generated a graphical model (Figure 5) that summarizes the relative positioning of fibroblasts, iPS-MSCs, and iPS cells along an epithelial–mesenchymal identity axis. The model integrates morphological features, EMT marker expression, CD44 isoform usage, and ESRP1 expression to map epithelial, intermediate epithelial/mesenchymal, and mesenchymal identity states in non-transformed human cells. Importantly, the diagram represents identity state mapping rather than experimental lineage progression. Together, this integrative framework illustrates how coordinated changes in splicing and transcriptional programs associate with epithelial, intermediate epithelial/mesenchymal, and mesenchymal identity states at the population level. The designation of the iPS-MSC state as intermediate is based on consistent population-level marker patterns and should not be interpreted as evidence of a uniform, stable single-cell hybrid EMT phenotype.

## 4. Discussion

In this study, we used a non-transformed cellular system composed of human foreskin fibroblasts, iPS-derived mesenchymal stem cells, and fully reprogrammed iPS cells to reconstruct a minimal and physiologically relevant epithelial–mesenchymal identity framework [19,20]. By integrating morphological observations, lineage marker profiling and targeted analysis of EMT-associated transcripts, we demonstrate that these three cell states reproducibly segregate into mesenchymal (fibroblasts), intermediate epithelial/mesenchymal (iPS-MSCs), and epithelial (iPS) identity states at the population level. Importantly, this distribution was reflected not only in classical EMT markers such as E-cad and VIM, but also in the architecture of CD44 isoform expression, a splicing-driven switch strongly implicated in cancer progression and metastasis [1,2,3]. Taken together, our results support the use of this simplified model as a tractable platform to dissect selected identity-associated molecular features to positioning along the E/M axis, rather than dynamic EMT/MET transitions.

A key finding of our study is the structured pattern of CD44 isoform usage that mirrors the hierarchical organization of cellular identity states. Fibroblasts predominantly expressed the standard CD44s isoform, consistent with their mesenchymal identity, whereas iPS cells exhibited broad upregulation of numerous epithelial-associated CD44 variant exons (v2–v10). iPS-MSCs occupied an intermediate position, characterized by partial inclusion of variant exons. These observations are consistent with extensive literature demonstrating that CD44 alternative splicing is a central determinant of cellular behavior, influencing processes such as adhesion, migration, receptor availability, and stemness [4,5,6,21]. In cancers, the transition from CD44v to CD44s is a well-established molecular hallmark of EMT and has been associated with increased invasiveness, metabolic adaptation, and metastatic potential [4,5]. By recapitulating this isoform distribution pattern in a non-malignant cellular system, our findings suggest that CD44 splicing is closely linked to fundamental programs of cell-state identity rather than being exclusively cancer-specific.

The intermediate behavior of iPS-MSCs is particularly notable. Although they are routinely classified as mesenchymal stem cells, their transcriptional and phenotypic properties have been shown to differ from those of tissue-derived MSCs. Several studies indicate that iPS-MSCs retain remnants of epithelial identity, including residual E-cadherin expression, activity of the miR-200 family, and partial repression of key EMT-associated transcription factors [7,8]. Our results support this concept: iPS-MSCs displayed moderate expression of E-cadherin and Vimentin, intermediate total CD44 levels and partial inclusion of multiple CD44 variant exons, collectively positioning them within an intermediate epithelial/mesenchymal identity at the population level. Intermediate epithelial/mesenchymal states have been proposed to combine epithelial and mesenchymal traits and have been associated with collective migration and tissue repair in specific biological contexts [3,9]. Thus, the iPS-MSC state may represent a useful surrogate for dissecting mechanisms specific to epithelial–mesenchymal plasticity rather than to terminal differentiation.

Although the present study focused primarily on CD44 alternative splicing and its regulation by ESRP1/2, the proposed model also enables the exploration of signaling pathways associated with epithelial–mesenchymal plasticity. ESRP1 and ESRP2 are well-established epithelial splicing regulators whose repression represents a hallmark of EMT, mediated by transcription factors such as TGF-β, ZEB1, and Snail [10,11,22].

Although both ESRP1 and ESRP2 exhibited expression patterns consistent with epithelial identity, ESRP1 was selected for functional perturbation due to its higher expression in epithelial iPS cells and its well-established dominant role in epithelial-specific alternative splicing. While potential compensatory or partially redundant functions of ESRP2 cannot be excluded, addressing such interactions would require combinatorial perturbation approaches beyond the scope of the present study.

In this context, analysis of selected Notch pathway components was included to provide broader signaling background [12,13]. However, in this non-transformed system, Notch-related transcriptional readouts did not parallel ESRP1 expression or CD44 isoform composition and were highest in iPS cells, consistent with reported roles of Notch signaling in pluripotency maintenance rather than in promoting mesenchymal identity [14,23]. These observations underscore the context-dependent configuration of EMT-associated regulatory pathways during physiological cell-state transitions.

Together, these findings indicate that while ESRP1/2 expression closely aligns with CD44 isoform selection along the epithelial–mesenchymal spectrum, Notch signaling represents an independent and context-dependent regulatory layer that does not directly parallel EMT-associated splicing changes in this model. This observation highlights that regulatory axes described in cancer may adopt distinct configurations during physiological cell-state transitions. An important implication of our findings is that non-malignant systems can capture selected molecular features associated with EMT and MET programs without the confounding effects of genomic instability and heterogeneous mutational backgrounds characteristic of cancer cells [1,15,24,25]. This distinction is particularly relevant in fields where EMT is often incorrectly equated with malignancy, rather than being recognized as a normal and highly regulated developmental and regenerative process. Importantly, epithelial–mesenchymal plasticity is not restricted to cancer but represents a fundamental biological process operating during embryonic development, tissue regeneration, wound healing, and fibrotic remodeling [1,15]. In these physiological contexts, transient or partial EMT-like programs enable cells to acquire migratory and adaptive properties without permanent loss of epithelial identity [1,2]. CD44 and its alternative splicing have been implicated in several non-malignant settings, including stem cell maintenance, tissue repair, and fibroblast activation, suggesting that regulation of CD44 isoform usage represents a general mechanism of cell-state plasticity rather than a cancer-specific phenomenon [5,6,7]. In light of this, the non-transformed system used here should be viewed primarily as a model of physiological epithelial–mesenchymal identity regulation, with relevance to cancer-associated EMT arising from shared underlying molecular programs rather than direct tumor-specific behavior [1,2].

This study also highlights several strengths of the proposed model. It is experimentally accessible, highly reproducible, and does not rely on genetic engineering. The defined identity states were robust and well-characterized at the morphological, phenotypic, and transcriptional levels. Moreover, because iPS cells are generated from fibroblasts and iPS-MSCs are subsequently derived from iPS cells, the system provides experimentally accessible mesenchymal, intermediate epithelial/mesenchymal, and epithelial identity states generated through defined reprogramming and differentiation procedures. This feature more closely mirrors identity changes observed during reprogramming, regeneration, and development than conventional immortalized cell lines [8,9]. In contrast to inducible cancer EMT–MET models based on transient growth factor stimulation (e.g., TGF-β exposure and withdrawal), the present system does not model dynamic EMT–MET transitions. Instead, it provides stable and experimentally accessible epithelial, intermediate, and mesenchymal identity states, enabling the analysis of identity-associated molecular programs independent of acute signaling perturbations.

Nevertheless, some limitations should be acknowledged. First, although the three examined cell populations clearly differ in their epithelial–mesenchymal (E/M) status, they represent discrete identity states rather than a continuous phenotypic landscape. Future studies employing single-cell transcriptomics approaches or time-resolved analyses during differentiation could provide a more refined view of intermediate identity states and transition dynamics. Second, CD44 alternative splicing is regulated by numerous signaling pathways beyond ESRP1/2 and Notch—including Wnt, FGFR2, Rbfox2, and hypoxia-associated pathways, which were not explored in the current experimental setting [10,16,26]. Dissecting the interplay between these regulatory axes will be important for a more comprehensive understanding of splicing-based epithelial–mesenchymal plasticity. Finally, functional assays such as migration, adhesion, or spheroid formation would further strengthen the causal link between molecular identity states and associated cellular behaviors. An additional limitation is the absence of protein-level validation of ESRP1 knockdown. Although ESRP1 silencing at the transcript level was efficient and consistently associated with altered CD44 splicing patterns, direct assessment of ESRP1 protein levels (e.g., by Western blotting) would be required to fully substantiate the extent of knockdown at the protein level. This analysis was beyond the scope of the present study and will be addressed in future work.

## 5. Conclusions

In conclusion, this study establishes a simple yet highly informative non-transformed human cell model that captures selected molecular features associated with epithelial–mesenchymal plasticity, including CD44 alternative splicing and its close association with cellular identity. By integrating primary dermal fibroblasts, iPS-derived mesenchymal stem cells, and fully reprogrammed iPS cells, the system provides experimentally accessible mesenchymal, intermediate epithelial/mesenchymal, and epithelial identity states positioned along an epithelial–mesenchymal identity axis. Importantly, our findings demonstrate that structured CD44 isoform distribution patterns and coordinated ESRP1/2 expression patterns reflect conserved aspects of epithelial–mesenchymal identity regulation beyond malignant contexts. As such, this model offers an experimentally tractable platform for dissecting identity-associated regulatory networks underlying epithelial–mesenchymal plasticity. Future studies integrating targeted ESRP1/2 perturbation, additional signaling pathways, and functional phenotypic assays will be required to elucidate how splicing-based and transcriptional programs interact to shape cellular plasticity during development, regeneration, and disease-associated processes.

## Data Availability

The original contributions presented in this study are included in the article and its Supplementary Materials. Further inquiries can be directed to the corresponding author.

## Supplementary Materials

The following supporting information can be downloaded at: https://www.mdpi.com/article/10.3390/cimb48020130/s1.

## Abbreviations

The following abbreviations are used in this manuscript:

ACTB	Beta-actin
CD44s	CD44 standard isoform
CD44v	CD44 variant isoforms
E-like	Epithelial-like
E/M	Epithelial/mesenchymal
E-cad	E-cadherin
EMT	Epithelial–mesenchymal transition
ESRP1	Epithelial splicing regulatory protein 1
ESRP2	Epithelial splicing regulatory protein 2
iPS cells	Induced pluripotent stem cells
iPS-MSCs	iPS-derived mesenchymal stem cells
M-like	Mesenchymal-like
MET	Mesenchymal–epithelial transition
MSCs	Mesenchymal stem cells
RT-qPCR	Reverse transcription quantitative polymerase chain reaction
SD	Standard deviation
siRNA	Small interfering RNA
VIM	Vimentin
