# Supplementary material for: ESRP1-Associated CD44 Alternative Splicing Stratifies Epithelial–Mesenchymal Identity States in a Non-Transformed Human Cell System
Source: Curr Issues Mol Biol. 2026 Jan 24;48(2):130. doi: 10.3390/cimb48020130 (PMC12939047; doi:10.3390/cimb48020130)
Supplement: Supplementary file 1 [file cimb-48-00130-s001.zip › cimb-4088821-supplementary.pdf]

Gene	Primer	Sequence
GAPDH	sense	5′- GCACCGTCAAGGCTGAGAAC -3′
	antisense	5′- ATGGTGGTGAAGACGCCAGT -3′
E-cadherin	sense	5′-GTCCTGGGCAGACTGAATTT-3′
	antisense	5′- GACCAAGAAATGGATCTGTGG -3′
ESRP1	sense	5′-ATAATCAGAGGCACAAACATCACAT-3′
	antisense	5′-ATAATAGAAACTGGGCTACCTCATTGG-3′
ESRP2	sense	5′-TGCCACAGAGGATGACTTTG-3′
	antisense	5′-ATTGACTGCTGGGCTCTTTG-3′
Vimentin	sense	5′-CGAGGAGAGCAGGATTTCTC-3′
	antisense	5′- GGTATCAACCAGAGGGAGTGA -3′
CD44_total	sense	5′-ATAATTGCCGCTTTGCAGGTGTATT-3′
	antisense	5′-ATAATGGCAAGGTGCTATTGAAAGCCT-3′
CD44s	sense	5′-ATAATAAAGGAGCAGCACTTCAGGA-3′
	antisense	5′-ATAATTGTGTCTTGGTCTCTGGTAGC-3′
CD44v2	sense	5′-ATAATCAGCAACTGAGACAGCAACCAA-3′
	antisense	5′-ATAATAACCAATCCCAGGTTTCTTGCC-3′
CD44v3	sense	5′-ATAATGGCTGGGAGCCAAATGAAGAAA-3′
	antisense	5′-ATAATCATCATCATCAATGCCTGATCCAGA-3′
CD44v4	sense	5′-ATAATCAGTGGAACCCAAGCCATTCAA-3′
	antisense	5′-ATAATCCTTGTGGTTGTCTGAAGTAGCAC-3′
CD44v5	sense	5′-ATAATGAAACTGGAACCCAGAAGCACA-3′
	antisense	5′-ATAATTGATGCTCATGGTGAATGAGGG-3′
CD44v6	sense	5′-ATAATCAGAAGGAACAGTGGTTTGGCA-3′
	antisense	5′-ATAATGTCTTCTTTGGGTGTTTGGCGA-3′
CD44v7	sense	5′-ATAATTGCAAGGAAGGACAACACCAAG-3′
	antisense	5′-ATAATGGGTGTGAGATTGGGTTGAAGA-3′
CD44v8	sense	5′-ATAATACGCTTCAGCCTACTGCAAA-3′

	antisense	5′-ATAATAAGAGGTCCTGTCCTGTCCAAA-3′
CD44v9	sense	5′-ATAATGAGCTTCTCTACATCACATGAAGGC-3′
	antisense	5′-ATAATGTCAGAGTAGAAGTTGTTGGATGGTC-3′
CD44v10	sense	5′-ATAATACCTCTCATTACCCACACACGA-3′
	antisense	5′-ATAATTAGCTGAGGTCACTGGGATGAA-3′
Notch1	sense	5′-CGCGCAGGGCCAGCAGATGAT-3′
	antisense	5′-GCACCCCACAGCCCACAAAGAACA-3′
Hes1	sense	5′-CCAGTTTGCTTTCCTCATTCC-3′
	antisense	5′-TCTTCTCTCCCAGTATTCAAGTTCC-3′
Hey1	sense	5′-TCTGAGCTGAGAAGGCTGGT-3′
	antisense	5′-CGAAATCCCAAACTCCGATA-3′
Hey2	sense	5′-AGATGCTTCAGGCAACAGGG-3′
	antisense	5′-CAAGAGCGTGTGCGTCAAAG-3′

Suppl. Table S1. Primers sequence for qRT-PCR.
